# Functional correlates of cognitive dysfunction in clinically isolated syndromes

**DOI:** 10.1371/journal.pone.0219590

**Published:** 2019-07-17

**Authors:** Sanuji Gajamange, Annie Shelton, Meaghan Clough, Owen White, Joanne Fielding, Scott Kolbe

**Affiliations:** 1 Department of Medicine and Radiology, University of Melbourne, Melbourne, Australia; 2 Department of Psychology, MIND Institute, and Center for Mind and Brain, University of California, Davis, Davis, California, United States of America; 3 Department of Neurosciences, Central Clinical School, Monash University, Melbourne, Australia; Charite Universitatsmedizin Berlin, GERMANY

## Abstract

Cognitive dysfunction can be identified in patients with clinically isolated syndromes suggestive of multiple sclerosis using ocular motor testing. This study aimed to identify the functional neural correlates of cognitive dysfunction in patients with clinically isolated syndrome using MRI. Eighteen patients with clinically isolated syndrome and 17 healthy controls were recruited. Subjects underwent standard neurological and neuropsychological testing. Subjects also underwent functional MRI (fMRI) during a cognitive ocular motor task, involving pro-saccade (direct gaze towards target) and anti-saccade (direct gaze away from target) trials. Ocular motor performance variables (averaged response time and error rate) were calculated for each subject. Patients showed a trend towards a greater rate of anti-saccade errors (*p* = 0.09) compared to controls. Compared to controls, patients exhibited increased activation in the right postcentral, right supramarginal gyrus, and the right parietal operculum during the anti-saccade>pro-saccade contrast. This study demonstrated that changes in functional organisation of cognitive brain networks is associated with subtle cognitive changes in patients with clinically isolated syndrome.

## Introduction

Multiple sclerosis is associated with a range of cognitive dysfunctions, affecting domains such as attention, working memory and information processing speed. While not all patients are overtly cognitively impaired, subtle cognitive changes may be evident at first presentation with a clinically isolated syndrome (CIS), becoming more pronounced as the disease progresses [1–3]. It is thus critical to identify assessment tools that can detect and predict the likely trajectory of cognitive decline in patients with CIS in order to optimise treatment at disease onset.

Clinically, cognitive dysfunctions in people with multiple sclerosis are commonly identified through self-reporting, which is subjective and unreliable. More recently, neuropsychological tests such as the paced auditory serial addition test (PASAT) and the symbol digit modalities test (SDMT) have been used as a tool for identifying cognitive dysfunction in patients with established diseases. However, these neuropsychological tests are relatively insensitive to subtle cognitive dysfunction [4, 5].

An alternative approach to assess cognition is with the use of a cognitive eye movement task. Involving saccadic responses to simple visual stimuli, eye movement tasks can probe several key components of cognition, including attention, memory, working memory and decision making, and can reveal dysfunctions of these processes in neurological diseases like multiple sclerosis [6].

Compared to healthy controls, multiple sclerosis participants, including CIS patients, typically exhibit increased response latencies, poorer spatial accuracy of initiating and correcting saccades, and a greater proportion of directional errors [4, 5, 7–10]. Critically, poor saccadic performance are evident in CIS patients in the absence of neuropsychological deficits, making ocular motor measures a useful method for testing early cognitive changes [4].

Magnetic resonance imaging (MRI) can provide information regarding structural and functional changes in the central nervous system that drive cognitive decline in people with CIS. To assess specific functional activity associated with cognition, a cognitive task is performed during a functional MRI (fMRI) scan. Using task-related fMRI, studies have reported changes in the topology and magnitude of brain activations during the performance of neuropsychological cognitive tasks [11–20], even in cohorts without overt cognitive dysfunction [12, 13].

This study aimed to better understand the relative contributions of changes in the brain to early cognitive dysfunction in CIS patients. We did this by evaluating cognitive variation and underlying functional substrates in CIS patients using MRI and cognitive assessments. Task-related fMRI changes in cognition were assessed using an eye movement task involving pro-saccade (direct gaze towards target) and anti-saccade (direct gaze away from target) trials. Unlike standard neuropsychological tests, ocular motor tasks require no verbal or body movement that can cause head motion during imaging. A specific focus on CIS patients allowed us to examine early changes to cognition and in the brain prior to overt neurodegeneration.

## Methods

### Participants

Eighteen patients with CIS (24–53 years, 15 females) were recruited from the Royal Melbourne Hospital, Australia. Inclusion was based on the initial neurological disturbance (ten patients presented with a visual disturbance, three with numbness/muscle weakness, two with headaches, one with balance problems, one with a disturbance in the brain stem and one with an unknown disturbance), with or without the presence of demyelination on MRI. All testing was performed no less than six months after initial presentation to reduce the influence of acute relapse on cognitive and imaging variables. All patients had normal visual acuity at the time of MRI scanning (6/6 or better in both eyes) (S1 Table). Seventeen healthy controls (26–54 years, 16 females) with no reported history of neurological disease were recruited for comparison.

This study was conducted in accordance with the Declaration of Helsinki and was approved by the Human Research Ethics Committee of Monash University, Australia. All study participants provided voluntary, written consent. No patients exhibited a severity of cognitive impairments such that it was not clear that they were not able to provide informed consent. This was determined by physicians and nurses based on their medical history and through consultation with the participant.

### Neuropsychological assessments

Working memory, attention, and information process speed were assessed using Paced Auditory Serial Addition (PASAT) [21] and Symbol Digit Modality Test (SDMT) [22]. For each task, a single score was calculated by four trained examiners. One patient declined to perform the PASAT task.

### Clinical MRI assessments

All MRI data was acquired using a 3T Magnetom Skyra (Siemens, Erlangen, Germany) scanner using a 20-channel receiver head coil. A 3D T1-weighted MPRAGE sequence (TR = 1540 ms, TE = 2.55 ms, a flip angle of 9°, FOV = 256 x 256 mm^2^ for a voxel size = 1 x 1 x 1 mm^3^) was collected for volumetric analyses. A 3D T2-weighted FLAIR sequence (TR = 5000 ms, TE = 387 ms, FOV = 230 mm for a voxel size = 0.8 x 0.8 mm^3^ in-plane and 0.9 mm^3^ sagittal-plane) was acquired for lesion identification and segmentation. Brain volume measures were calculated with FreeSurfer (v1.75.2.2) using the standard cross-sectional analysis pipeline [23] and expressed as fractions of intracranial volume. Ventricle size, corpus callosum, cerebellum, cortical grey and white matter, subcortical grey matter (thalamus, caudate, accumbens, putamen, globus pallidus and ventral diencephalon) were examined. Lesions were segmented using MRIcro (v1.40) (www.cabi.gatech.edu/mricro/mricro/).

### Ocular motor task fMRI

**Fig 1:**
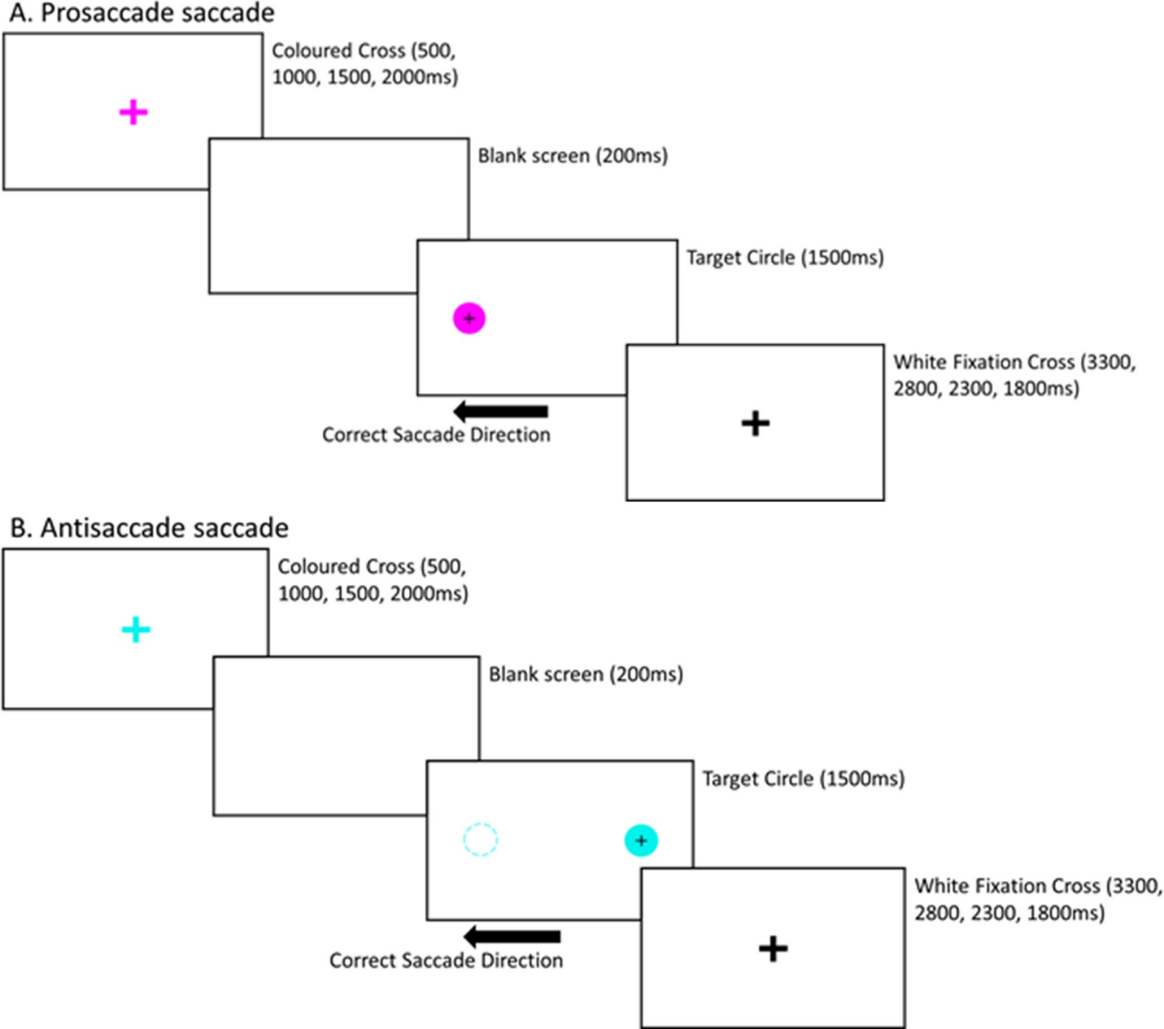
Schematic diagram of the interleaved prosaccade and antisaccade ocular motor paradigm performed during the functional MRI. The colour of the fixation cross indicates whether the trial is A. a pro-saccade (directing gaze towards the target) or B. an anti-saccade (diverting gaze away from the target–note, the dashed circle does not appear on the screen).

Participants performed an interleaved ocular motor switch task during fMRI acquisition (Fig 1). The task consisted of 96 pro-saccade, 96 anti-saccade, and 28 null trials within a single fMRI block. Specifically, the task involved performing pro-saccades (PS) (directing gaze to the target) and anti-saccades (AS) (directing gaze away from the target, towards the opposite direction) as accurately and quickly as possible. Pro- and anti-saccade trials were presented in pseudorandomised order across four blocks. Each trial began with a central fixation cross (96 x 96 pixels, white) that was presented for a variable amount of time (500–2000 ms) on a black background. The cross was followed by a blank screen (200 ms), after which a target stimulus (filled circle; diameter = 96 pixels) appeared on either the left or right side of the cross. The colour of the cross (magenta/turquoise) indicated the trial types (PS/AS) with the colour/trial type combinations pseudorandomised. The target remained for 1500 ms, during this time the participant made either a PS or AS. Immediately after the target disappeared, a second fixation cross (96 x 96 pixels, white) appeared until the end of the trial (duration varied as a function of initial fixation duration). Each PS and AS trial lasted for a total of 5500 ms. Null trials consisted of a white fixation cross, presented for 3500 ms.

Horizontal displacement of the eye was recorded using a MR-compatible infrared video eye tracking system (EyeLink 1000, SR Research, Canada) with a spatial resolution of 0.01 degrees and a sampling speed of 500 Hz. Customised software programmed in MATLAB was used to identify the timing and gaze parameters for each saccade, and to compare timing and gaze data to stimulus timing and position. A criterion of ≥ 30°/s was used to define saccade onset. Trials were excluded if they exhibited 1) blinks, 2) unstable or poor fixation prior to the saccade or 3) anticipatory saccades, defined as saccades made within 100 ms of the target circle appearing. Anticipatory saccades were not examined due to reduced number of said saccades. Each trial was examined for directional errors, defined as looking at the target circle on AS trials and looking away from the target circle on PS trials. Ocular-motor measures, 1) saccade latency, defined as the time difference between target circle onset and saccade onset and 2) directional error rate, defined as the proportion of the trials incorrectly executed, were calculated for both pro- and anti-saccade trials [4, 8].

Functional MRI was performed using a T2*-weighted echo-planar imaging sequence (TR = 2500 ms, TE = 30 ms, FOV = 192 mm, acquisition matrix = 64 x 64 x 64 for a voxel size = 3 x 3 x 3 mm^3^). A total of four blocks were collected, each with 133 volumes and 44 axial slices to capture the whole brain (5 mins and 42 sec per run). Analysis of fMRI task data was performed using fMRI Expert Analysis Tool (FEAT), part of the FMRIB's software library (FSL v5.0.4, www.fmrib.ox.ac.uk). Pre-processing for each fMRI run involved: (1) slice timing correction, (2) motion correction using rigid body transformations (MCFLIRT), (3) removal of non-brain tissue, (4) spatial smoothing with a non-linear smoothing algorithm (SUSAN, [24], with an extent threshold of 6 × 6 × 6 mm^3^), and (5) high-pass nonlinear temporal filtering with cut-off of 1/50 Hz. FMRIB's Linear Registration Tool (FLIRT) was then used to register the functional data to the T1-weighted image and then to the standard MNI 2 x 2 x 2 mm^3^ template with a 12 degrees-of freedom affine transformation.

After pre-processing and registration, fMRI data for each run were analysed for the contrasts (1) AS>PS and (2) PS>AS. A second level analysis using FSL fixed effects statistical modelling was conducted to create subject-level contrast maps across all four experimental blocks. Finally, a third level analysis using FMRIB's Local Analysis of Mixed Effects (FSL FLAME) was performed to determine the difference between patients and healthy controls (CIS>controls and controls>CIS), corrected for age and sex, as well as the main effect across patients and controls. Significance maps were family-wise error corrected using a cluster-extent threshold of z>2.3 and *p*  =  0.05.

**Fig 2:**
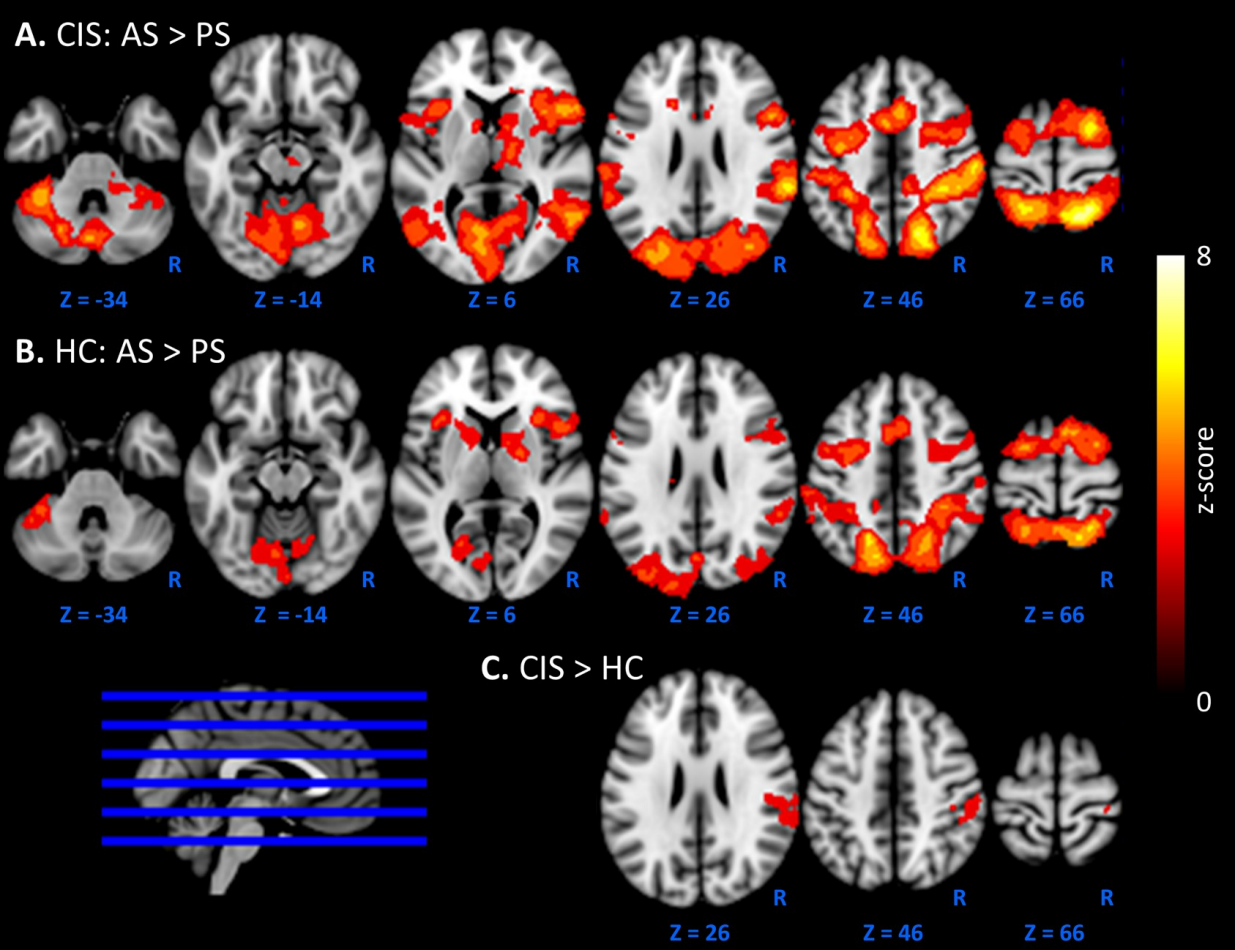
Brain activation during antisaccade (AS)>prosaccade (PS) in CIS patients. The main effect for **A.** CIS patients and **B.** healthy control (HC) subjects, coloured by z-score are overlayed on a MNI 2mm standard brain. **C.** Areas of significant increase of activations in the right lateral parietal of patients compared to controls).

The maximum z-score for each region of interest (ROI) within the AS>PS and PS>AS networks was extracted for correlation analyses (S1 Fig). Firstly, the main effect maps of AS>PS and AS<PS were thresholded at *Z* = 4 to identify ROIs (Fig 2). Each ROI was parcellated into non-contiguous regions, and those with a minimum of 100 voxels were retained. A threshold of Z = 4 was used to provide a clear separation between brain activation regions, and 100 voxels ensured that large robust brain activation regions were assessed. Parcellation maps of 7 cerebral networks by [25] were used to classify the AS>PS and PS>AS networks. The AS>PS network consisted of 11 ROIs and overlapped with the dorsal attention network (34.7% voxel overlap), visual network (21.3% voxel overlap), ventral attention network (12.7% voxel overlap), frontoparietal network (6.6% voxel overlap), somatomotor network (3.5% voxel overlap) and default mode network (1.0% voxel overlap). The PS>AS network consisted of eight ROIs and overlapped with the default mode network (88.6% voxel overlap), limbic network (4.0% voxel overlap) and frontoparietal network (1.4% voxel overlap). Note, early visual cortex regions were removed from the PS>AS network as these regions are associated with visual presentation. Furthermore, the ROI labelled as right subcortical region included the caudate (17.8%), thalamus (14.8%) and pallidum (9.9%) according to the Harvard-Oxford subcortical structural atlas in FSL.

### Statistical analyses

The following statistical analyses were performed. For all tests, normality was tested using Kolmogorov-Smirnov. For non-normally distributed data, rank tests were performed if transformation of the data (logarithmic or polynomial) did not normalise the distribution. Significance level was set at *p*<0.05 with multiple comparison corrections applied where appropriate according to the specific methods described below.

Behavioural measures (ocular motor measures and neuropsychological test scores) and volumetric measures (ventricle size, corpus callosum, cerebellum, cortical and subcortical grey matter, and white matter) were compared between CIS patients and healthy controls using independent samples t-test (for equal or unequal variances) or Mann-Whitney U (if the assumption for normality was violated). In CIS patients, Spearman rank-order correlation analyses were performed to determine the relationship between ocular motor measures (AS latency and directional error rate) and neuropsychological test scores (PASAT and SDMT).

In patients and controls, a post-hoc analysis was also performed to examine the relationship between level of activation and ocular motor performance. Regression analyses were performed between subject-wise z-score for each ROI within the AS>PS network and AS measures (latency and directional error rate), controlling for age and gender. Regression analyses were performed to determine the relationship between the average z-score across the AS>PS and AS<PS networks and (1) lesion volume, and (2) volumetric measures (ventricle size, corpus callosum, cerebellum, cortical and subcortical grey matter, and white matter), controlling for age and gender.

## Results

### Demographics, disease and cognition

Table 1 summarises demographic and disease characteristics for patients and controls. Compared to controls, people with CIS did not differ in age, years of education, brain volumetric measures or any behavioural parameters, and showed a trend towards a greater proportion of AS directional errors (*p* = 0.087).

**Table 1:** Demographics, disease characteristics. Volumetric, neuropsychological scores and ocular motor measures for patients and control subjects.

		**CIS** **(n = 18)**	**Controls (n = 17)**		
		*Mean (SD)*	*Mean (SD)*	*Effect size*	*p-value*
***Demographics***					
Age (yrs)		36.50 (10.19)	38.71(9.45)	-0.227	0.51
Education (yrs)		16.36 (2.86)	15.88 (3.20)	0.160	0.64
***Radiological***					
Lesion volume (cm^3^)		3.14 (3.32)	-	-	-
Ventricle fraction (x10^-3^)		10.21 (5.56)	10.52 (4.02)	-0.065	0.66
Corpus callosum fraction (x10^-3^)		2.30 (0.53)	2.38 (0.31)	-0.171	0.62
Cerebellum fraction		0.102 (0.010)	0.103 (0.011)	-0.100	0.77
Cortical WM fraction		0.340 (0.032)	0.342 (0.027)	-0.082	0.81
Cortical GM fraction ^*a*^		0.425 (0.038)	0.430 (0.039)	-0.110	0.59
Subcortical GM fraction (x10^-3^)		30.04 (3.21)	30.59 (2.25)	-0.200	0.56
***Neuropsychological***					
*PASAT* ^*b*^					
	Correct (%)	78.92 (18.16)	86.27 (11.31)	-0.479	0.17
*SDMT*					
	Correct (no.)	59.56 (10.79)	64.18 (9.79)	-0.433	0.19
*Ocular motor*					
*Pro-saccade*					
	Latency (ms)	264.23 (52.99)	251.85 (57.31)	0.226	0.44
	Directional error rate (%) ^a^	3.94 (3.40)	2.63 (3.06)	0.399	0.23
*Anti-saccade*					
	Latency (ms)	331.05 (55.66)	330.26 (61.29)	0.014	0.97
	Directional error rate (%)	9.26 (6.59)	5.94 (4.31)	0.575	0.087

^**a**^ Independent samples Mann-Whitney U Test.

^**b**^ 17 CIS patients included.

WM = white matter, GM = grey matter, PASAT = paced auditory serial addition test, SDMT = symbol digit modalities test.

Table 2 summarises the correlation between cognitive ocular motor and neuropsychological variables in patients. Anti-saccade latency correlated with PASAT (ρ = -0.75, *p* = 0.47 x 10^−3^, S2 Fig) and AS directional error rate correlated with SDMT scores (ρ = -0.48, *p* = 0.043, S2 Fig). PS latency correlated with AS latency (r = 0.83, *p* = 0.02 x 10^−3^), and PS error rate correlated with AS error rate (ρ = 0.81, *p* = 0.04 × 10^−3^). Neither PS nor AS latencies correlated with their corresponding PS (ρ = 0.01, *p* = 0.96) or AS (r = -0.06, *p* = 0.80) error rates respectively.

**Table 2:** Spearman correlation between anti-saccade measures (latency and directional error rate) and neuropsychological scores (PASAT, SDMT) in patients. PASAT = paced auditory serial addition test, SDMT = symbol digit modalities test.

	Anti-saccade				
	Latency (ms)		Directional error rate (%)	
	ρ	*p*	ρ	*p*
PASAT	**-0.75**	**0.47 x 10**^**−3**^	0.12	0.66
SDMT	-0.37	0.13	**-0.48**	**0.043**

### Task-related functional activation

The pattern of activation for AS>PS is presented for patients (Fig 2A) and healthy controls (Fig 2B). Patients exhibited significantly stronger activation than controls during AS>PS, specifically in the right postcentral and supramarginal (anterior and posterior) gyrus, and the right parietal operculum (Fig 2C). The maximum *Z*-score of this significant cluster was 3.83, *p* = 0.011, and positioned at x = 40 mm, y = -30 mm, z = 56 mm, in MNI space. Controls did not exhibit significantly more activation than CIS patients in any brain regions. See S3 Fig for the effect size map for CIS>HC.

Table 3 summarises the relationship between activation level and ocular motor performance (AS latency and directional error rate) for each AS>PS network ROI in patients and controls. Control subjects exhibited significant correlations between activation level and AS latency across most ROIs whereas no correlation was detected for CIS patients.

**Table 3:** Association between activation across the AS>PS network and anti-saccade measures (latency and directional error rate) in patients and controls. DLPFC = dorsal lateral prefrontal cortex, FEF = frontal eye field, SEF = supplementary eye field.

			CIS		Controls	
			*β*	*p*	*β*	*p*
**Latency**						
	DLPFC		-0.09	0.76	-0.28	0.30
	Left FEF+SEF		-0.25	0.35	**-0.60**	**0.018**
	Right FEF+SEF		-0.38	0.17	**-0.65**	**0.008**
	Left frontal operculum		-0.08	0.77	-0.41	0.14
	Right frontal operculum		-0.28	0.34	**-0.80**	**0.003**
	Right subcortical		-0.27	0.32	**-0.78**	0.001*
	Left parietal		-0.02	0.95	-0.40	0.13
	Right parietal		-0.26	0.34	**-0.54**	**0.039**
	Left cerebellar VI		-0.05	0.87	-0.51	0.066
	Right cerebellar VI		-0.30	0.28	**-0.62**	**0.044**
	Left cerebellar IX		-0.30	0.27	-0.56	0.082
**Directional error rate**						
	DLPFC		-0.16	0.49	0.23	0.38
	Left FEF+SEF		-0.03	0.91	0.02	0.95
	Right FEF+SEF		0.13	0.59	0.16	0.55
	Left frontal operculum		0.02	0.95	-0.23	0.39
	Right frontal operculum		0.23	0.35	0.51	0.070
	Right subcortical		-0.09	0.70	0.35	0.19
	Left parietal		-0.21	0.39	0.18	0.48
	Right parietal		-0.20	0.39	0.19	0.47
	Left cerebellar VI		-0.34	0.18	0.38	0.15
	Right cerebellar VI		-0.02	0.93	0.03	0.93
	Left cerebellar IX		-0.23	0.33	0.50	0.10

* = survived multiple comparison using FDR.

The average level of activation within the AS>PS network and AS<PS network was correlated with lesion volume, volumetric and microstructural measures. In patients, greater lesion volume (β = -0.74, *p* = 0.001) and corpus callosum atrophy were associated with decreased AS>PS network activation (β = 0.52, *p* = 0.042), and corpus callosum atrophy was associated with increased AS<PS deactivation (β = 0.53, *p* = 0.014) (S2 Table).

## Discussion

This study investigated neural changes associated with cognitive function in people with clinically isolated syndrome indicative of multiple sclerosis. Using f MRI, we examined the early functional processes of cognitive dysfunction in CIS, assessed using an ocular motor task performed in the scanner. We found that functional activation was increased during the cognitive task in patients compared to controls, with activation levels lower in patients with greater pathological burden (lesion volume and atrophy). These results provide further evidence for functional neural plasticity in CIS patients that could play a role in mediating the effect of neuronal injury on cognitive decline.

### Ocular motor tasks as a tool for studying cognition in CIS

We examined cognitive function using an ocular motor task employing interleaved anti- and pro-saccades that could be performed in the MRI scanner without extra head motion. Compared to control subjects, CIS patients in this study showed no cognitive deficits at a group level (trend in AS directional error rate), demonstrating the subtlety of cognitive dysfunctions at the initial stage of the disease. Previous studies have reported significant cognitive dysfunction in CIS patients using ocular motor tasks outside the MRI scanner and with slightly larger cohort sizes [4, 5, 10]. Despite the lack of a significant group effect, we did observe significant correlations between saccade variables and neuropsychological scores, supporting the relevance of saccade variables to commonly measured cognitive domains [4, 5, 7, 9]. Specifically, increased AS response latencies were associated with PASAT scores. This is sensible given that the PASAT interrogates information processing speed, working memory and attention. In addition, we found that increased AS directional errors were associated with poorer performance on SDMT tasks that interrogate information processing speed. Although neuropsychological tests have recently been used to screen cognitive dysfunction, they can be stressful for patients, suffer from practice effects and rater bias and do not consistently identify subtle cognitive deficits in CIS cohorts [4, 26, 27]. Here, we have shown that a simple and objective MRI-compatible ocular motor task can be used as a proxy for a range of standard neuropsychological tests.

### Cognition and task-related activation

Overall, the pattern of activation exhibited when participants performed AS and PS trials is consistent with previous fMRI and positron emission tomography studies [28–34]. Compared to controls, patients exhibited greater functional activation during the cognitively more demanding AS condition compared to the simpler, reflexive PS condition in the right postcentral and supramarginal gyrus, and the right parietal operculum. Activation in the postcentral gyrus and the parietal operculum are associated with somatosensory processing [35] and activation within the supramarginal gyrus and medial frontal eye fields have been reported during saccade inhibition and attentional processing [36]. The involvement of these regions suggests that CIS patients require stronger saccade inhibition to perform anti-saccades correctly. Similar to the current study, several studies have reported enhanced activation in CIS [13–15] and those who developed multiple sclerosis [11, 16, 18, 37] patients compared to controls during various neuropsychological cognitive tasks. However, results from these studies need to be interpreted with caution, as vocal responses required for the PASAT and SDMT can introduce stimulus correlated head motion that is known to cause fMRI artefacts. That said, this enhanced activation in patients is interpreted as a compensatory mechanism to preserve cognitive function [11, 13]. Interestingly, we found patients with greater lesion load and corpus callosum atrophy, displayed reduced activation within the AS>PS network, despite overall increased in activation in CIS patients compared to controls. Similarly, a study in early multiple sclerosis (6.7 ± 4.94 month disease duration) reported an inverse correlation between task-based activation during the PASAT and normal appearing white matter injury, despite greater task-based activation in patients compared to controls [38]. We also examined the relationship between levels of activation in the AS>PS and AS<PS networks and cognitive performance. In patients, neither AS latency nor error rate correlated with brain activation. In contrast, healthy controls showed a stronger association between increased activation and shorter AS latencies. The absence of this relationship in patients provides further evidence for aberrant neural activity during cognitive ocular motor performance.

### Methodological considerations

Similar to neuropsychological tests, ocular motor tasks are also influenced by disease induced sensory motor deficits. However, patients in this study did not exhibit increased saccade latency, and unlike controls, saccade latency did not correlate with functional activation. This suggests that processing speed is not impaired in patients, and instead patients are processing information differently to controls.

Rather than data-driven approaches commonly used for studying brain connectivity, we have used task-related fMRI to identify ocular motor networks (AS>PS and AS<PS). As such, we were able to examine functional activation within the same networks of brain regions.

This study recruited only a relatively small cohort of patients commensurate with inherent difficulties in recruiting people with CIS. Furthermore, longitudinal studies are required to elucidate the change in brain organisation with disease progression.

## Conclusion

This work examined the functional correlates of cognitive function in CIS patients. Despite a lack of overt cognitive dysfunction in patients, we observed increased functional activation during cognitive task performance. These results suggest that changes in functional organisation of cognitive brain networks underlie subtle cognitive dysfunction in people with CIS.

## Supporting information

S1 Table Ocular motor measures (pro- and anti-saccade) did not differ significantly between controls, CIS patients with a history of optic neuritis (ON) and CIS patients without a history of ON. (PDF)

S2 Table Association between functional activation and brain injury in patients. (PDF)

S1 Fig Regions of interest derived from the switch ocular motor task. (PDF)

S2 Fig Scatterplots show the relationships between anti-saccade (AS) and neuropsychological measures in patients. (PDF)

S3 Fig Effect size of functional activation for CIS>healthy controls during antisaccade (AS)>prosaccade (PS) contrast. (PDF)

S1 Data Spreadsheet containing all data. (SAV)

## Abbreviations

AS: anti-saccade
CIS: clinically isolated syndrome
fMRI: functional MRI
PASAT: Paced Auditory Serial Addition
PS: pro-saccade
ROI: region of interest
SDMT: Symbol Digit Modality Test
