# Supplementary material for: Functional correlates of cognitive dysfunction in clinically isolated syndromes
Source: PLoS One. 2019 Jul 17;14(7):e0219590. doi: 10.1371/journal.pone.0219590 (PMC6636738; doi:10.1371/journal.pone.0219590)
Supplement: S1 Table — (PDF) [file pone.0219590.s001.pdf]

S1 Table. *Ocular motor measures (pro- and anti-saccade) did not differ significantly between controls, CIS patients with a history of optic neuritis (ON) and CIS patients without a history of ON.*

	Healthy control (n = 17)	*CIS with a history of ON (n = 10)*	*CIS without a history of ON (n = 8)*
Prosaccade			
Latency (ms)	251.8 ± 57.3	279.1 ± 60.5	245.7 ± 37.6
Directional error (%)	2.6 ± 3.1	4.5 ± 3.5	3.3 ± 3.4
Antisaccade			
Latency (ms)	330.3 ± 61.3	341.5 ± 49.2	318.0 ± 63.7
Directional error (%)	5.9 ± 4.3	7.2 ± 7.2	5.4 ± 5.4
