# Supplementary material for: Functional correlates of cognitive dysfunction in clinically isolated syndromes
Source: PLoS One. 2019 Jul 17;14(7):e0219590. doi: 10.1371/journal.pone.0219590 (PMC6636738; doi:10.1371/journal.pone.0219590)
Supplement: S2 Table — (PDF) [file pone.0219590.s002.pdf]

S2 Table. Association between functional activation and brain injury in patients. *WM = white matter; GM = grey matter. * = survived multiple comparison using FDR*

	β	p
**AS>PS network activation**		
Lesion volume	**-0.74**	**0.001***
Ventricle fraction	-0.21	0.51
Corpus callosum fraction	**0.52**	**0.042**
Cerebellum fraction	0.14	0.64
Cortical WM fraction	0.36	0.17
Cortical GM fraction	-0.05	0.90
Subcortical GM fraction	0.49	0.11
**AS<PS network activation**		
Lesion volume	-0.32	0.15
Ventricle fraction	-0.32	0.26
Corpus callosum fraction	**0.53**	**0.014**
Cerebellum fraction	-0.16	0.55
Cortical WM fraction	0.41	0.064
Cortical GM fraction	-0.04	0.90
Subcortical GM fraction	0.39	0.15
