# Supplementary material for: Functional correlates of cognitive dysfunction in clinically isolated syndromes
Source: PLoS One. 2019 Jul 17;14(7):e0219590. doi: 10.1371/journal.pone.0219590 (PMC6636738; doi:10.1371/journal.pone.0219590)
Supplement: S1 Fig — (PDF) [file pone.0219590.s003.pdf]

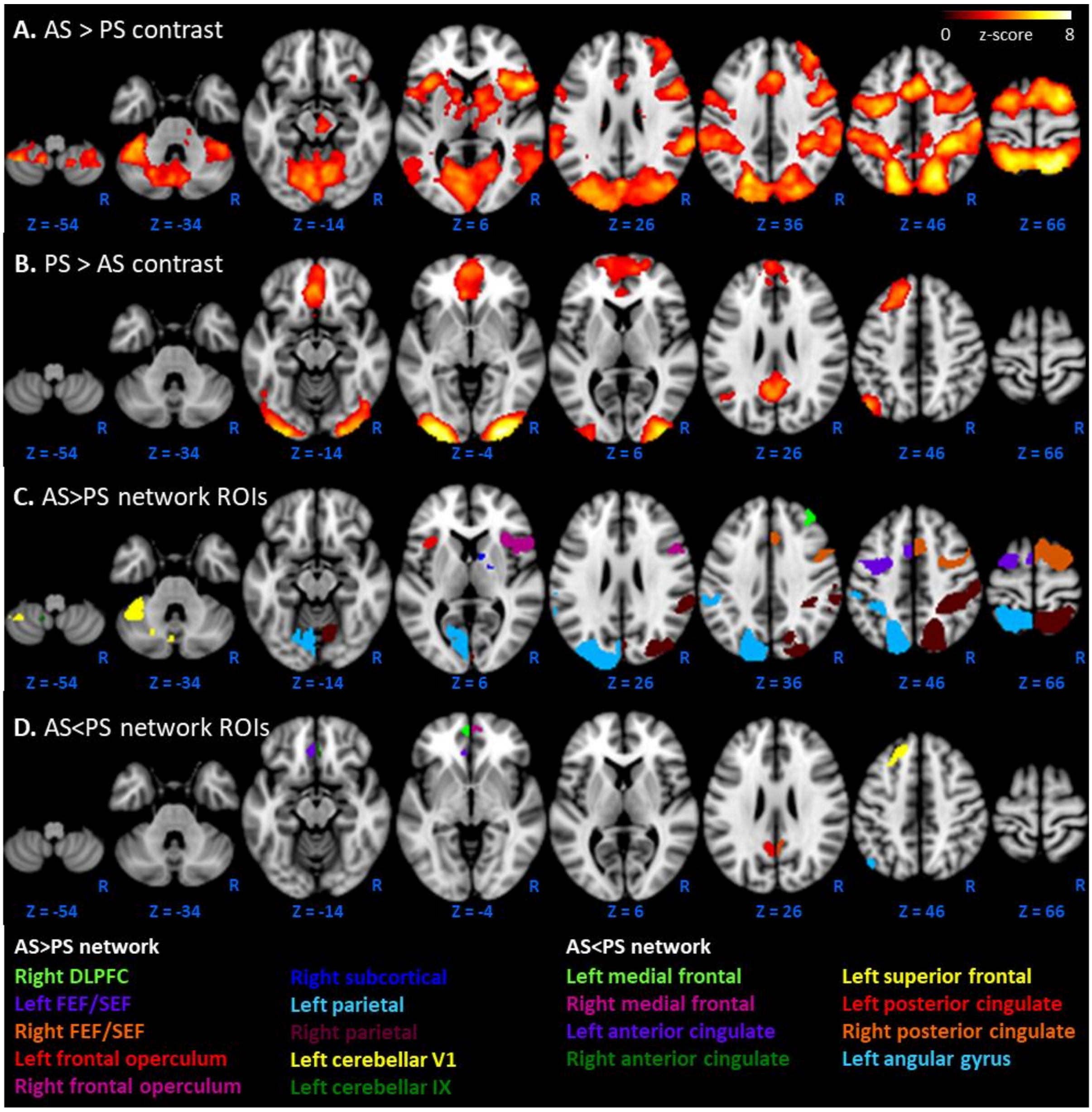

S1 Fig. Regions of interest derived from the switch ocular motor task. *The anti-saccade (AS) vs prosaccade (PS) contrasts were used to derive regions of interests (ROI). Significant regions of the main effect of* ***A.*** *AS>PS network and* ***B.*** *AS<PS network across both cohorts were parcellated into regions. DLPFC = dorsal lateral prefrontal cortex, FEF = frontal eye fields, SEF = supplementary eye fields,*
