# Supplementary material for: Functional correlates of cognitive dysfunction in clinically isolated syndromes
Source: PLoS One. 2019 Jul 17;14(7):e0219590. doi: 10.1371/journal.pone.0219590 (PMC6636738; doi:10.1371/journal.pone.0219590)
Supplement: S2 Fig — (PDF) [file pone.0219590.s004.pdf]

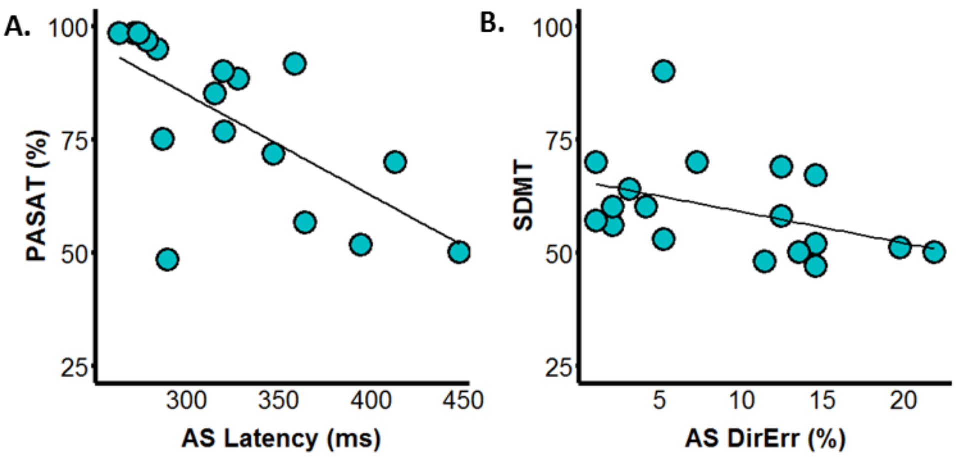

S2 Fig. Scatterplots show the relationships between anti-saccade (AS) and neuropsychological measures in patients. *AS latency correlated with paced auditory serial addition test (PASAT) scores, and AS directional error rate correlated with symbol digit modalities test (SDMT) scores*
