# Supplementary material for: Functional correlates of cognitive dysfunction in clinically isolated syndromes
Source: PLoS One. 2019 Jul 17;14(7):e0219590. doi: 10.1371/journal.pone.0219590 (PMC6636738; doi:10.1371/journal.pone.0219590)
Supplement: S3 Fig — (PDF) [file pone.0219590.s005.pdf]

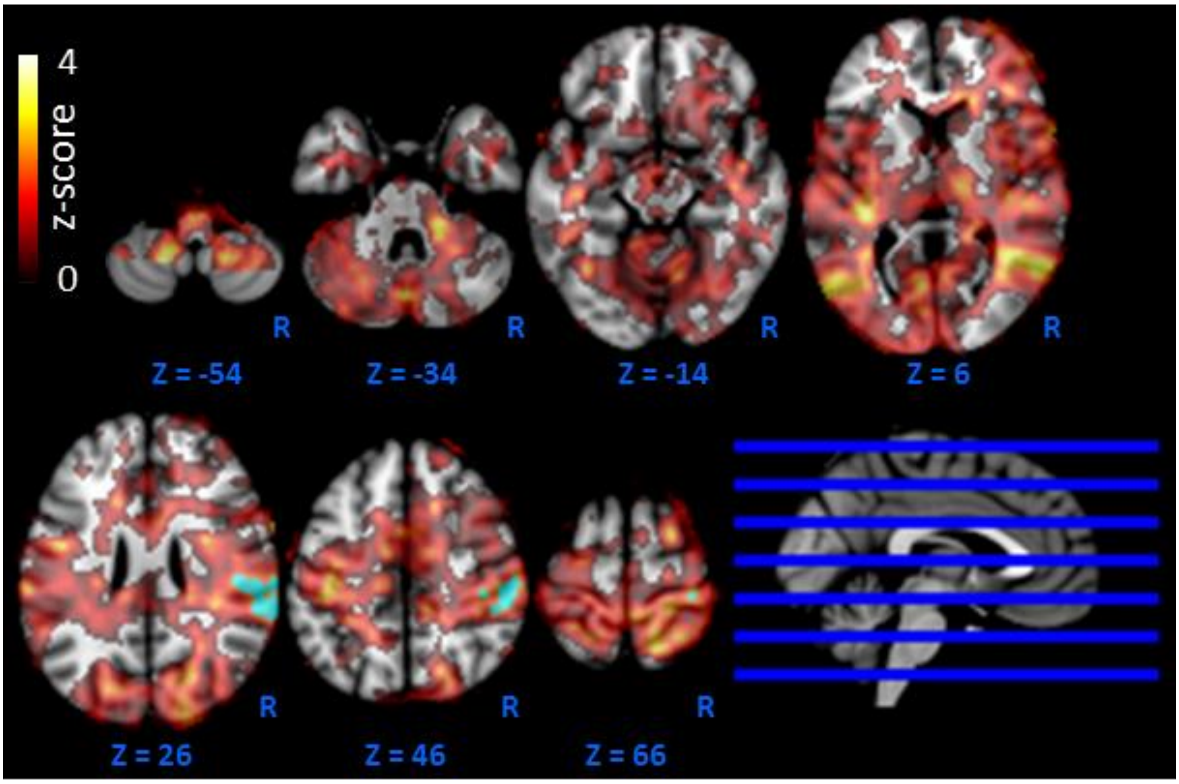

S3 Fig. Effect size of functional activation for CIS>healthy controls during antisaccade (AS)>prosaccade (PS) contrast. *Z-scores for CIS>healthy control subjects overlapped over significant group-specific region (blue) to show widespread functional differences between patients and controls*
